# Exploring the role of class stigma in socioeconomic inequalities in type 2 diabetes: The Maastricht Study

**DOI:** 10.1177/13591053251395856

**Published:** 2026-01-13

**Authors:** Bengisu Sezer, Jeroen Albers, Rachelle Meisters, Miranda T. Schram, Bastiaan E. de Galan, Carla van der Kallen, Marleen van Greevenbroek, Pieter Dagnelie, Annemarie Koster, Hans Bosma

**Affiliations:** 1 Department of Social Medicine, Care and Public Health Research Institute (CAPHRI), Maastricht University, Maastricht, Netherlands; 2 Department of Internal Medicine, Maastricht University Medical Center+, CARIM School for Cardiovascular Diseases, Maastricht University, Maastricht, the Netherlands; 3 MHeNS School for Mental Health and Neuroscience, Heart and Vascular Center, Maastricht University Medical Center (MUMC+), Maastricht University, Maastricht, the Netherlands; 4 Department of Epidemiology, Erasmus MC, Rotterdam, the Netherlands; 5 Department of Internal Medicine, Radboud University Medical Center, Nijmegen, the Netherlands

**Keywords:** classism, socioeconomic inequalities, stigma, type 2 diabetes

## Abstract

Studying class stigma in the context of diabetes and socioeconomic inequalities may shed light on the societal factors influencing diabetes. Data from 1947 participants aged 49–88 who participated in the second phase of The Maastricht Study were used. SEP (education, income, occupation) and a six-item classism scale were measured through self-reported questionnaires. Prediabetes and T2D were defined through an oral glucose tolerance test. We conducted multinomial logistic regression analyses to investigate the associations between SEP, classism, and diabetes. About 20.9% (*N* = 406) of the study sample had T2D (Mean_age_ = 69.8 ± 6.8; 31.3% women). People with low SEP had T2D (e.g. income OR = 2.13, 95% CI: 1.54–2.82) and reported perceived classism (e.g. education OR = 2.07, 95% CI: 1.33–3.21) more often than people with high SEP. Classism was not statistically significantly associated with T2D. Social health inequalities are apparent in T2D; however, our results suggest that class stigma might not be a major factor in the underlying processes. Further research should investigate chronic stress and the intersection of stigmas.

## Introduction

Low socioeconomic position (SEP) is a risk factor for type 2 diabetes (T2D; Brown et al., 2004; Hill-Briggs et al., 2020). People with a lower SEP more often have adverse environmental conditions (Brulle and Pellow, 2006). Such environmental conditions might be the cause of poor health through stress exposure (Cundiff et al., 2020) or through health behaviours (Pampel et al., 2010). Stigmatisation and discrimination also play crucial roles in the societal environment of low SEP. One of the factors that might increase the chronic stress levels or hinder health behaviours is class stigma that people with low SEP experience.

Stigmatisation can be one of the pathways through which socioeconomic health inequalities might emerge and persist (Clouston and Link, 2021; Krieger, 2014). People from lower social classes are more likely to experience feelings of powerlessness (Bosma, 2006) and inferiority (Simons et al., 2017), social inadequacy (Bosma et al., 2015), or status anxiety (Crielaard et al., 2023) as a result of social comparison. Being part of a lower social class might cause status anxiety or perceived class stigma. Class stigma or classism can be described as prejudice based on someone’s social class (Pincus and Sokoloff, 2008) and is more commonly observed in people with low SEP (Simons et al., 2018). People with low SEP might feel looked down due to their social class, causing high levels of perceived class stigma. Social conditions in one’s environment are embodied and internalised (Krieger, 2005). Internalised negative social perceptions may cause detrimental health consequences through self-stigmatisation (Hatzenbuehler et al., 2013).

Stigma has substantial health consequences through limited access to resources, social isolation, dysfunctional psychological and behavioural responses, and chronic stress (Hatzenbuehler et al., 2013). For example, people who experience social stigma receive less social support due to social isolation (Kawachi and Berkman, 2001). Perceived classism has many negative health outcomes including decreased psychological well-being (Inglis et al., 2023), self-rated health (Simons et al., 2017), and health-related behaviours (Ahuja et al., 2022). While these studies demonstrated the association between class stigma and other health outcomes, class stigma is not yet extensively researched within the context of diabetes. A previous study demonstrated the mediating effect of status anxiety in socioeconomic inequalities in T2D (Crielaard et al., 2023). Although status anxiety is a similar construct to class stigma, the authors did not study the perceived classism leaving a gap in the T2D research. This type of stigma can be one of the many psychosocial mechanisms that explains the ways SEP influences health (Inglis et al., 2023). Being a target of stigmatisation might impair health either directly, through stress-related neuroendocrine responses which result in poor health outcomes (Bosma, 2006), or indirectly, through behavioural pathways such as poor coping mechanisms with stress (Paradies, 2006). The evidence on the association between class stigma and diabetes is limited and requires further investigation.

People with a low SEP have a higher risk for diabetes (Hill-Briggs et al., 2020), while they are also more vulnerable to class stigma. Considering class stigma is more commonly present in low SEP, it might be one of the stress-inducing neuroendocrine or immunological mechanisms that explains socioeconomic inequalities in health as well as an influence on health-related behaviours. It is important to study stigmatisation in the context of prediabetes and T2D to understand the societal exposome of diabetes as well as to develop effective interventions that target decreasing class stigma or dealing with self-stigmatisation in health-related contexts. Therefore, we will explore the ways class stigma contributes to the explanation of socioeconomic inequalities in diabetes. In the present study, we aim to understand if stigmatisation is a pathway in the association of low SEP and T2D. We hypothesise that people with a low SEP have T2D and report perceived class stigma more often than people with a high SEP, and people who report class stigma have T2D more often than those who do not. Existing literature demonstrates the association of age and sex with SEP and diabetes status (Hu et al., 2025; Maty et al., 2008). Since they can be important covariates in this association, all statistical models will be adjusted for age and sex.

## Method

### Study population

We used data from The Maastricht Study, an observational prospective population-based cohort study. The rationale and methodology have been described previously (Schram et al., 2014). In brief, the study focuses on the aetiology, pathophysiology, complications, and comorbidities of T2D and is characterised by an extensive phenotyping approach. Eligible participants were aged between 40 and 75 years and living in the southern part of the Netherlands. Participants were recruited through mass media campaigns and from the municipal registries and the regional Diabetes Patient Registry by mailings reaching to 9187 participants in the final cohort. Recruitment was stratified according to known T2D status with an oversampling of individuals with T2D for reasons of efficiency. The first phase of the data collection took place between September 2010 and October 2020 within a period of 3 months per participant. The study was approved by the institutional ethics committee and all participants gave written informed consent.

A second phase of measurements started in November 2020 and is still ongoing. For the current study, we used cross-sectional data from 2567 participants who completed the second phase measurement between November 2020 and April 2023 within the course of 3 months. The final sample consisted of 1947 participants after the exclusion of people with diabetes other than T2D, and people with missing values in education and classism scale. Additionally, people with missing values in SEP indicators were excluded for a complete case analysis for each SEP model of education, income (*N* = 1627), and occupation (*N* = 1759). The exclusion steps are illustrated in Figure S1.

## Measurements

### Diabetes status

Prediabetes and prevalent T2D were defined in accordance with WHO 2006 criteria (WHO, International Diabetes Federation, 2006). All participants underwent a standardised seven-point oral glucose tolerance test after overnight fasting. Blood samples were collected at baseline and 15, 30, 45, 60, 90, and 120 minutes after the consumption of a 75 g glucose drink. Participants who were insulin-dependent or with a fasting glucose level higher than 11.0 mmol/l (as determined by finger prick) did not undergo this test. The glucose levels were categorised as normal glucose levels, prediabetes, and T2D. Prediabetes was defined as impaired fasting glucose (fasting plasma glucose 6.1–7.0 mmol/l and 2-hour plasma glucose < 7.8 mmol/l) or impaired glucose tolerance (fasting plasma glucose < 7.0 mmol/l and 2-hour plasma glucose ⩾ 7.0–11.1 mmol/l). T2D was defined as fasting plasma glucose ⩾ 7.0 mmol/l or 2-hour plasma glucose ⩾ 11.1 mmol/l. Participants on diabetes medication and without type 1 diabetes were also considered to have T2D.

## Socioeconomic position

### Education

Participants were asked to indicate the highest education level they had completed in categories (1 = None, 2 = Primary educational level, 3 = Lower vocational education, 4 = Intermediate general secondary education, 5 = Intermediate vocational education, 6 = Higher general secondary education, 7 = Higher vocational education, 8 = University education, 9 = Uncompleted primary educational level, 10 = Other, 11 = I don’t know) during the first phase of data collection. Categories 10 (Other) and 11 (do not know) were coded as missing values. ‘Uncompleted primary education (9)’ was recoded as the second answer category (2). After recoding, we categorised education level as low (1–5), intermediate (6–7), and high (8–9) education, approximately creating tertiles.

### Occupation

Participants were asked to describe their current occupation in an open-ended question during the first phase of data collection. The open-ended question was coded to International Standard Classification of Occupations 2008 (ISCO-08) categories by a trained coder (Ganzeboom, 2010; Organisation for Economic Co-operation and Development (OECD), 2011). Subsequently, the ISCO codes were converted to the International Socio-Economic Index of Occupational Status (ISEI-08) to indicate occupational class status forming a continuous scale. The continuous scale reflects occupational status on a scale from 10 (e.g. farmers) to 89 (e.g. medical practitioners). We categorised the ISEI-08 classifications as low, intermediate, and high occupation based on tertiles.

### Income

Participants were asked to indicate their net monthly household income in 19 categories ranging from ‘less than 750 euros’ to ‘more than 5000 euros’. Then, the midpoint of the reported household income was divided by the square root of household size to achieve an equivalent value per person (OECD, 2011, 2012). We categorised the final income as low, intermediate, and high income based on tertiles.

### Classism

Class stigma was measured through an eight-item questionnaire (Mickelson and Williams, 2008). Participants reported the extent to which they agreed or disagreed with each statement on a five-point Likert scale from definitely disagree (1) to definitely agree (5; e.g.: ‘I feel strange or abnormal because of my financial situation, level of education, or profession’.; Cronbach’s α = 0.58). Two positively stated items showed smaller correlations with the other items and loaded into a different factor (e.g. ‘I never feel shy when I am in public’.). Thus, we excluded these items from our analysis and used the six-item version of the scale (Cronbach’s α = 0.86). Based on the criteria of Simons and colleagues (Simons et al., 2017), we categorised people reporting four or five in at least one of the six items as ‘perceiving classism’ (*N* = 140, 7.2%).

### Statistical analysis

Descriptive statistics were computed across T2D status and class stigma levels. By conducting multinomial logistic regression analysis, we investigated the separate relationships of SEP with class stigma, and of SEP and class stigma with diabetes status. The analyses were conducted for each SEP indicator separately. All assumptions of multinomial logistic regression were met in our models. All models were adjusted for age in years and sex in two categories. We initially planned to conduct a mediation analysis; however, because of a lack of statistically significant association between classism and diabetes, a mediation analysis was not conducted. Instead, we included classism as a covariate in the models of SEP and diabetes status to observe the association between SEP and diabetes status independent of classism. For sensitivity analyses, we explored the interaction of age and sex with SEP and class stigma, and class stigma and SEP while predicting diabetes status. We also conducted all logistic regression analyses by using continuous variables of SEP indicators and classism to test the robustness of results compared to categorical analyses. All analyses were conducted in the statistical software, RStudio (Team R, 2015) by using *tidyverse* (Wickham and Wickham, 2019) for data cleaning and manipulation, and *nnet* (Ripley et al., 2016) for logistic regression analysis.

## Results

In the study sample, participants were aged between 49 and 88 (Mean_age_ = 67.6, SD_age_ = 7.6). 20.9% (*N* = 406) of the participants had T2D (*M*_age_ = 69.8(±6.8); 31.3% women). Table 1 presents the distribution of SEP indicators over T2D status. People with a low SEP more often had T2D than people with a high SEP. For example, 18.0% of people with high education had T2D compared to 26.9% of people with low education. About 16.6% of people with a high income had prediabetes, compared to 17.0% of people with a low income. However, this association was not the same for occupation as 16.2% of people with a high occupation had prediabetes unlike 15.4% of people with a low occupation. Table S1 also presents the distribution of study characteristics across perceived classism levels. 12.0% of people with a low income reported perceived classism compared to 4.9% of people with a high income.

**Table 1.** Distribution of age, sex, and socioeconomic position by diabetes status.

Variables of Interest	Normal glucose metabolism	Prediabetes	T2D	*p*-Value
Age *M*(SD)	66.4 (7.66)	69.4 (7.09)	69.8 (6.84)	<0.001
Sex				<0.001
Female	676 (71.4%)	144 (15.2%)	127 (13.4%)	
Male	542 (54.2%)	179 (17.9%)	279 (27.9%)	
Education *M*(SD)	6.9 (1.56)	6.67 (1.65)	6.42 (1.67)	0.001
High	597 (66.4%)	140 (15.6%)	162 (18.0%)	
Intermediate	357 (62.4%)	99 (17.3%)	116 (20.3%)	
Low	264 (55.5%)	84 (17.6%)	128 (26.9%)	
Occupation *M*(SD)	57.4 (17.6)	56.4 (18.1)	52.6 (19.6)	<0.001
High	459 (66.4%)	112 (16.2%)	120 (17.4%)	
Intermediate	425 (65.7%)	107 (16.5%)	115 (17.8%)	
Low	239 (56.8%)	65 (15.4%)	117 (27.8%)	
Income *M*(SD)	2601 (896)	2537 (845)	2322 (836)	<0.001
High	363 (68.6%)	88 (16.6%)	78 (14.7%)	
Intermediate	339 (62.9%)	86 (16.0%)	114 (21.2%)	
Low	311 (55.6%)	95 (17.0%)	153 (27.4%)	

*Note*. Means (and standard deviations) and *p*-values for ANOVA tests are reported for continuous variables and number of people (and row percentages) and *p*-values for chi-squared tests are reported for categorical variables.

Table 2 illustrates the association between SEP and classism. The results show that people with lower levels of education and income more often reported perceived classism than those with higher levels of education and income (e.g. education OR = 2.07, 95% CI: 1.33–3.21). The association between occupational status and classism were in the same direction; however, it was not statistically significant (OR = 1.56, 95% CI: 0.98–2.49). The intermediate level SEP did not statistically significantly differ from the high SEP level in terms of perceived classism. Table 3 shows the association between perceived classism and diabetes status. Our results showed that self-reported class stigma was neither associated with prediabetes (OR = 0.97, 95% CI: 0.59–1.60) nor with T2D (OR = 0.78, 95% CI: 0.48–1.29).

**Table 2.** The association of SEP and classism.

SEP indicators	Classism binary
	High
	OR (95% CI)
Education^a^	0.88* (0.79–0.98)
Education std^b^	0.81* (0.68–0.96)
High	Ref
Intermediate	1.21 (0.80–1.85)
Low	2.07*** (1.33–3.21)
Occupation^a^	1.00 (1.00–1.00)
Occupation std^b^	0.86 (0.72–1.03)
High	Ref
Intermediate	1.16 (0.75–1.80)
Low	1.56 (0.98-2.49)
Income^a^ (per 100e)	0.94*** (1.00–1.00)
Income std^b^	0.58*** (0.48–0.71)
High	Ref
Intermediate	1.39 (0.81–2.39)
Low	3.43*** (2.12–5.57)

*Note*. The reference category is low classism. Models are adjusted for age and sex. Separate models were used for each SEP indicator.

a Education scale ranges from 1 to 9, occupation ranges from 10 to 89, income scale ranges from 600e to 6000e.

b *Z*-standardised scores.

* *p* < 0.05. **p < 0.01. ***p < 0.001.

**Table 3.** The association of classism with diabetes status.

		Prediabetes	T2D
Classism	*N*	OR (95% CI)	OR (95% CI)
Classism binary			
No perceived classism	1807	Ref	
Perceived classism	140	0.97 (0.59–1.60)	0.78 (0.48–1.29)

*Note*. The reference category is normal glucose metabolism. Models are adjusted for age and sex.

* *p* < 0.05. **p < 0.01. ***p < 0.001.

Table 4 presents the association between SEP levels and pre(diabetes) when adjusted for age and sex. People with a lower level of education were more likely to have T2D than people with a high education (OR = 1.69, 95% CI: 1.27–2.26). People with an intermediate level of income were also more likely to have T2D than people with a high income (OR = 1.45, 95% CI: 1.04–2.02). The association of education and occupation with prediabetes was in the same direction as T2D; however, not statistically significant. When adjusted for classism in Model 2, low SEP remained statistically significantly associated with prediabetes and T2D.

**Table 4.** The association of SEP with prediabetes and T2D.

	Model 1			Model 2	
		Prediabetes	T2D	Prediabetes	T2D
SEP indicators	N	OR (95% CI)	OR (95% CI)	OR (95% CI)	OR (95% CI)
Education^a^		0.94 (0.087–1.01)	0.84*** (0.79–0.91)	0.93 (0.86–1.01)	0.84*** (0.78–0.90)
Education std^b^		0.90 (0.79–1.02)	0.76*** (0.68–0.85)	0.90 (0.79–1.02)	0.76*** (0.67–0.85)
High	900	Ref			
Intermediate	572	1.27 (0.95–1.71)	1.32 (1.00–1.74)	1.27 (0.95–1.71)	1.32 (1.00–1.75)
Low	476	1.19 (0.87–1.64)	1.69*** (1.27–2.26)	1.20 (0.87–1.64)	1.72***(1.29–2.30)
Occupation^a^		1.00 (1.00–1.00)	1.00*** (1.00–1.00)	1.00 (1.00–1.00)	1.00*** (1.00–1.00)
Occupation std^b^		0.92 (0.80–1.05)	0.72*** (0.63–0.81)	0.92 (0.80–1.05)	0.71*** (0.63–0.81)
High	692	Ref			
Intermediate	647	1.12 (0.83–1.51)	1.19 (0.89–1.61)	1.12 (0.83–1.52)	1.20 (0.89–1.61)
Low	421	1.14 (0.81–1.62)	2.07*** (1.52–2.82)	1.14 (0.80–1.62)	2.08*** (1.53–2.84)
Income^a^ (per 100e)		1.00 (0.98–1.01)	0.96*** (0.95–0.98)	1.00 (0.98–1.01)	0.96*** (0.95–0.98)
Income std^b^		0.98 (0.86–1.13)	0.73*** (0.63–0.83)	0.98 (0.85–1.13)	0.71*** (0.62–0.82)
High	529	Ref			
Intermediate	539	0.92 (0.66–1.29)	1.45* (1.04–2.02)	0.92 (0.66–1.29)	1.45* (1.04–2.03)
Low	559	1.06 (0.75–1.48)	2.13*** (1.54–2.95)	1.06 (0.75–1.49)	2.19*** (1.58–3.03)

*Note*. Model 1 is adjusted for age and sex. Model 2 is adjusted for age, sex, and classism. Separate models were used for each SEP indicator.

a Education scale ranges from 1 to 9, occupation ranges from 10 to 89, income scale ranges from 600e to 6000e.

b *Z*-standardised scores.

* *p* < 0.05. **p < 0.01. ***p < 0.001.

The interaction between SEP and classism and the interaction of classism with age or sex were not statistically significantly associated with T2D. The interaction between low level of education and age in years in relation to T2D was statistically significant, but not any other SEP indicator. We have further investigated this interaction term by conducting simple slope analysis. In younger ages, higher education was associated with lower odds of T2D, while this effect was not statistically significant for older ages.

We have also investigated the missingness patterns and reported the number of missing values in each variable in Table S2. The results of the Little’s MCAR test did not support missing completely at random (χ²(8) = 74.8, *p* < 0.05). To assess the robustness of our findings, we have also conducted the analysis with multiple imputation. After imputing 20 datasets and pooling the results, the relationship between SEP and classism as shown in Table S3, classism and diabetes status as shown in Table S4, and SEP and diabetes status as shown in Table S5 demonstrated comparable results to the complete case analyses.

## Discussion

This study aimed to understand socioeconomic health inequalities in diabetes and the role of classism in this association. We investigated whether class stigma was associated with SEP and diabetes status, and whether it was a pathway in SEP and diabetes association. First, as expected, people with low SEP across all SEP indicators more often had prediabetes and T2D. Second, people with low SEP in education and income reported more perceived classism than people with high SEP. Third, contrary to our expectations, class stigma was not statistically significantly associated with diabetes status. Thus, our results indicate that classism might not be a crucial factor in explaining socioeconomic health inequalities in diabetes in our sample.

Meritocratic beliefs contain the idea that an individual’s success is based on their individual efforts, thus promoting a sense of deservingness for those who are in the lower end of social hierarchy (Simons et al., 2017). As a result, people with a low SEP are perceived negatively and stigmatised due to their social position. Supporting this view, we found that people with a low SEP more often reported perceived classism. We found this association for education and for income levels, but not for occupational status. Considering their older age, most of our participants were no longer working. So, it is possible that stigmas around the job status are no longer relevant for this group. Out of three SEP indicators, education was observed to be the most robustly associated with both classism and diabetes status. It is important to note that all three SEP indicators assess different aspects of SEP (Geyer et al., 2006), and education is the most stable indicator throughout the life course (Zajacova and Lawrence, 2018) which might have caused its stronger association with perceived classism. Perceived classism should be studied in the context of health inequalities to understand the psychological and social factors in this relationship. The social determinants of T2D are complex (Hill-Briggs et al., 2020). Studying the social context that T2D emerges can help deconstruct the complex pathways between SEP and T2D which eventually contribute to better prevention, treatment, and intervention strategies.

Although people with low SEP more often felt looked down at, which probably affected their mental health negatively (Inglis et al., 2023), we did not find any statistically significant association between perceived classism and T2D. Because of a lack of significant association, we did not conduct counterfactual mediation analysis. Instead, we presented the models controlled for classism in model 2. While previous studies reported the negative health effects of classism on several health outcomes such as mental health (Mickelson and Williams, 2008), self-rated health (Simons et al., 2017), and health-related behaviours (Ahuja et al., 2022), in our data, there was no such association with glucose metabolism status. Our findings are at odds with a previous study demonstrating the role of status anxiety in diabetes (Crielaard et al., 2023). One of the factors that could potentially explain the discrepancy between our results and existing literature can be the operationalisation of classism, which differs across studies. For example, Crielaard and colleagues measured status anxiety as feeling inferior due to one’s social class (Crielaard et al., 2023). Although they are similar constructs, feeling inferior differs from perceived classism as perceived classism refers to the perception of being looked down by others due to one’s social class while feeling inferior refers to an upwards social comparison. It is possible that our study sample did not internalise class stigma (Hatzenbuehler et al., 2013). Stigmatisation might impair health through stress exposure (Cundiff et al., 2020) or through health behaviours (Pampel et al., 2010). Future research can study the associations of perceived classism and stress responses or health behaviours to understand the role of classism better. Another potential explanation could be the lack of diversity in our sample in terms of ethnicity, immigration status, or experiences of classism. The majority of our participants were Western European, living in a relatively homogenous environment. It is possible that the effects of class stigma are more prominent when other stigmatising characteristics are also present. In a previous ecological study, the Netherlands was found to rank lower on people’s perceptions of classism (Layte and Whelan, 2014) with only 8.6% of the people agreeing with the statement ‘others look down on me because of my job situation or income’. Nevertheless, other studies using a Dutch sample found that people with low SEP experienced class stigma (Simons et al., 2017, 2018). Although perceived classism might still be an issue for some subgroups in the Netherlands, it is possible that overall, our dataset represented a sample where perceived classism and social comparison did not vary enough to observe adverse health outcomes. Lastly, it is also possible that stress-related causes of poor health are more relevant for health outcomes such as cardiovascular diseases since their direct association with cardiovascular disease was more commonly showed in the literature, but the stress and T2D were mostly studied indirectly (Joseph and Golden, 2014; Osborne et al., 2020). As a result of the lack of an association between classism and T2D, we also did not test the mediating effects of classism in the association of SEP and T2D.

The study has several strengths in its research design. We used data from a large cohort study, which allowed us to perform analyses in a large sample. Another strength of our study is the operationalisation of class stigma. Previous studies vary in their ways of describing classism and often use single-item measurements. We extended the definition of classism to feelings of inferiority due to one’s social class and financial status by using a validated six-item scale to measure perceived class stigma. The scale is a valid measure of assessing perceived class stigma (Cannella, 2020) and showed good reliability. Furthermore, our line of research helps destigmatising diabetes as a lifestyle disease and shifts the attention to socioeconomic and environmental factors by emphasising the social gradient in diabetes.

It is important to acknowledge the limitations of our study. First, we cannot draw causal conclusions from our results due to the cross-sectional design of the study; however, previous research in our sample showed the longitudinal association between SEP and diabetes status (Sezer et al., 2023). Second, education and occupation were measured during the first phase of the data collection, approximately 10 years before the measurement point of classism and diabetes status. While this might be a limitation, we did not expect large changes in education and occupation considering that the majority of our sample was no longer working. The highest level of education also did not change between two phases of the study. Moreover, people with T2D were oversampled in the first phase of the study. This implies that the prevalence of people with T2D in our study sample does not reflect the prevalence of T2D in the Netherlands. Additionally, the patterns of the missing data did not hold the missing completely at random assumption. Our analyses relied on the missing at random assumption; however, this cannot be tested statistically. We tackled this limitation by demonstrating that the results from the complete case analyses and the analyses on the imputed datasets were similar. Lastly, our study sample lacked diversity in terms of ethnicity and class. Our sample included mostly Western European participants with higher education levels, in an older aged group. Future research can consider broader age groups and intersectional identities such as race, gender, class, and presence of multiple stigmas such as weight and class stigmas.

Our research shows the socioeconomic inequalities in T2D and the link between socioeconomic status and perceived class stigma. People with lower education and income reported higher perceived classism. This finding suggests the importance of the social context in sharing health inequalities. By understanding the societal context in which T2D emerges, we can develop more effective interventions. Treatment, intervention, and prevention strategies should consider the different psychosocial profiles of people with a low SEP. Qualitative research can help us understand the experiences of people from disadvantaged backgrounds. Although perceived classism was more common among people with low SEP, class stigma might not be a major target in explaining socioeconomic pathways in diabetes in our study sample. The underlying factors behind socioeconomic health disparities are complex. Social and psychological environment in low SEP should be investigated carefully. Future research can investigate other societal factors potentially mediating the association of SEP and T2D such as chronic stress or intersectional identities.

## Supplemental Material

sj-docx-1-hpq-10.1177_13591053251395856 – Supplemental material for Exploring the role of class stigma in socioeconomic inequalities in type 2 diabetes: The Maastricht Study Supplemental material, sj-docx-1-hpq-10.1177_13591053251395856 for Exploring the role of class stigma in socioeconomic inequalities in type 2 diabetes: The Maastricht Study by Bengisu Sezer, Jeroen Albers, Rachelle Meisters, Miranda T. Schram, Bastiaan E. de Galan, Carla van der Kallen, Marleen van Greevenbroek, Pieter Dagnelie, Annemarie Koster and Hans Bosma in Journal of Health Psychology
