# Supplementary material for: Exploring the role of class stigma in socioeconomic inequalities in type 2 diabetes: The Maastricht Study
Source: J Health Psychol. 2026 Jan 13;31(9):3578–88. doi: 10.1177/13591053251395856 (PMC13365321; doi:10.1177/13591053251395856)
Supplement: sj-docx-1-hpq-10.1177_13591053251395856 – Supplemental material for Exploring the role of class stigma in socioeconomic inequalities in type 2 diabetes: The Maastricht Study [file sj-docx-1-hpq-10.1177_13591053251395856.docx]

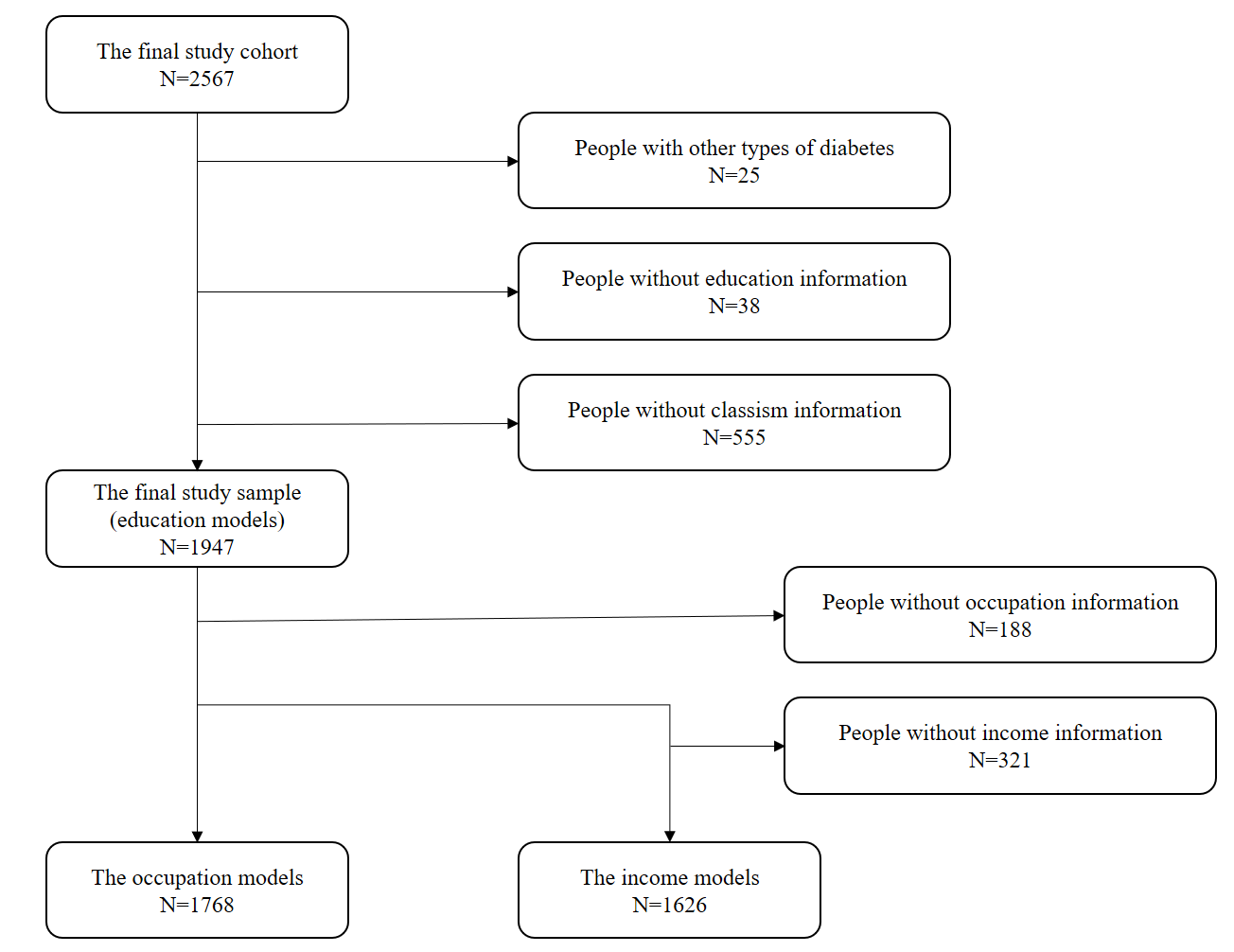


Figure 1S. Exclusion criteria

| Table S1. Descriptive statistics across classism levels | | |  |
| --- | --- | --- | --- |
|  | Classism Binary | |  |
|  | No Perceived Classism | Perceived Classism | *p-*value |
| Age *M(SD)* | 67.9(7.47) | 64(7.88) | <.001 |
| Sex |  |  | .261 |
| Female | 872(92.1%) | 75(7.9%) |  |
| Male | 935(93.5%) | 65(6.5%) |  |
| Education |  |  | .135 |
| High | 845(94.0%) | 54(6.0%) |  |
| Intermediate | 528(92.3%) | 44(7.7%) |  |
| Low | 434(91.2%) | 42(8.8%) |  |
| Occupation |  |  | .231 |
| High | 649(93.9%) | 42(6.1%) |  |
| Intermediate | 599(92.6%) | 48(7.4%) |  |
| Low | 384(91.2%) | 37(8.8%) |  |
| Income |  |  | <.001 |
| High | 503(95.1%) | 26(4.9%) |  |
| Intermediate | 508(94.2%) | 31(5.8%) |  |
| Low | 492(88.0%) | 67(12.0%) |  |
| Diabetes Status |  |  | .132 |
| Normal Glucose | 1120(92.0%) | 98(8.0%) |  |
| Prediabetes | 302(93.5%) | 21(6.5%) |  |
| T2DM | 385(94.8%) | 21(5.2%) |  |
|  | | |  |

Table S2. Missing values in exposures and mediators (N=2542).

|  | **Missing values** |
| --- | --- |
| **Age** | - |
| **Sex** | - |
| **Diabetes status** | - |
| **Education** | 38 (1.49%) |
| **Occupation** | 291 (11.4%) |
| **Income** | 458 (18.01%) |
| **Classism** | 567 (22.31%) |

| Table S3. The association of SEP and classism after multiple imputation, N=2542 | | |
| --- | --- | --- |
|  | Classism Binary | |
|  | | High |
|  | | OR (95%CI) |
| Education | | 0.88*  (0.79-0.97) |
| High | | ref |
| Intermediate | | 1.08  (0.72-1.63) |
| Low | | 1.70*  (1.12-2.59) |
| Occupation | | 0.99  (0.98-1.00) |
| High | | ref |
| Intermediate | | 1.08  (0.70-1.65) |
| Low | | 1.44  (0.91-2.26) |
| Income (per 100e) | | 0.95***  (0.93-0.97) |
| High | | ref |
| Intermediate | | 1.35  (0.86-2.13) |
| Low | | 2.39***  (1.52-3.76) |
| *p <.05, **p<.01, ***p<.001  Note. The reference category is low classism. Models are adjusted for age and sex.  Note. Separate models were used for each SEP indicator. | | |

| Table S4. The association of classism with diabetes status after multiple imputation, N=2542. | | | |  |  |  |
| --- | --- | --- | --- | --- | --- | --- |
|  |  | Prediabetes | T2D |  |  |  |
|  | N | OR (95%CI) | OR (95%CI) |  |  |  |
| Classism binary |  |  |  |  |  |  |
| No perceived classism |  | ref |  |  |  |  |
| Perceived classism |  | 0.93  (0.59-1.48) | 0.83  (0.53-1.31) |  |  |  |
| *p <.05, **p<.01, ***p<.001  Note. The reference category is normal glucose metabolism. Models are adjusted for age and sex. | | | |  |  |  |
| Table S5. The association of SEP with prediabetes and T2D after multiple imputation, N=2542. | | | | | | |
|  | | Model 1 | | | Model 2 | |
|  | |  | Prediabetes | T2D | Prediabetes | T2D |
|  | | N | OR (95%CI) | OR (95%CI) | OR (95%CI) | OR (95%CI) |
| Education | |  | 0.95  (0.89-1.02) | 0.81***  (0.77-0.87) | 0.95  (0.89-1.02) | 0.81***  (0.76-0.86) |
| High | |  | ref |  |  |  |
| Intermediate | |  | 1.22  (0.93-1.59) | 1.53***  (1.19-1.96) | 1.22  (0.93-1.59) | 1.55***  (1.21-1.98) |
| Low | |  | 1.13  (0.86-1.49) | 1.92***  (1.50-2.46) | 1.12  (0.85-1.48) | 1.92***  (1.50-2.46) |
| Occupation | |  | 1.00  (0.99-1.00) | 0.98***  (0.98-0.99) | 0.99  (0.99-1.00) | 0.98***  (0.98-0.99) |
| High | |  | ref |  |  |  |
| Intermediate | |  | 1.13  (0.87-1.48) | 1.19  (0.92-1.52) | 1.14  (0.86-1.50) | 1.17  (0.91-1.52) |
| Low | |  | 1.14  (0.84-1.55) | 2.12***  (1.63-2.75) | 1.14  (0.84-1.55) | 2.12***  (1.63-2.77) |
| Income (per 100e) | |  | 1.00  (0.98-1.01) | 0.97***  (0.95-0.98) | 1.00  (0.98-1.01) | 0.97***  (0.95-0.98) |
| High | |  | ref |  |  |  |
| Intermediate | |  | 0.96  (0.71-1.31) | 1.32  (0.99-1.76) | 0.95  (0.71-1.27) | 1.36*  (1.02-1.82) |
| Low | |  | 1.05  (0.79-1.40) | 1.96***  (1.49-2.59) | 1.05  (0.78-1.42) | 2.08***  (1.56-2.78) |
| *p <.05, **p<.01, ***p<.001  Note. Model 1 is adjusted for age and sex. Model 2 is adjusted for age, sex, and classism.  Note. Separate models were used for each SEP indicator. | | | | | | |
